# Systemic Activity and Phytotoxicity of Fluensulfone in Vegetable Transplants Infected by *Meloidogyne incognita*

**DOI:** 10.2478/jofnem-2025-0036

**Published:** 2025-08-31

**Authors:** Francisco Franco-Navarro, Antoon T. Ploeg

**Affiliations:** Department of Nematology, University of California Riverside, 3401 Watkins Drive, Riverside, CA 92521, U.S.A.

**Keywords:** bell pepper, eggplant, leaf spray application, melon, non-fumigant nematicide, Southern Root-Knot Nematode, tomato

## Abstract

Fluensulfone is the active ingredient of the non-fumigant nematicide Nimitz. It is much less harmful to the environment and has much improved worker safety compared to broad-spectrum fumigant nematicides. The product is registered for use in a variety of crops, including fruiting vegetables, and is applied to soil 7–14 days before seeding or planting. Although labeled for soil application, earlier research suggested that fluensulfone has systemic nematicidal activity when applied as a leaf spray application but also may cause some phytotoxic effects in some crops when applied as a leaf spray. In replicated greenhouse pot experiments, the nematicidal activity and phytotoxicity of fluensulfone applied as a soil drench was compared to a leaf spray application in tomato, eggplant, melon, and three pepper cultivars. A leaf spray application with fluensulfone significantly reduced *Meloidogyne incognita* infestation of the roots but was highly phytotoxic to melon and eggplant. Tomato and particularly peppers were less sensitive to fluensulfone leaf sprays. In further experiments we showed that the active ingredient is exuded or leaks out of the roots into the rhizosphere as spraying tomato or pepper plants with fluensulfone resulted in a high mortality of *M. incognita* second-stage juveniles in a water suspension surrounding the roots. This effect was observed within 12–24 hr after the spray application.

Root-knot nematodes (RKN), *Meloidogyne* spp., are the most important plant-parasitic nematodes infecting bell pepper (*Capsicum annuum*) worldwide (Di Vito et al., 1985; Di Vito et al., 1992; Thies and Fery, 2000; Thies et al., 2008). Although the genus *Meloidogyne* contains nearly 100 species, only five species are considered as major plant-parasitic nematodes of economic importance (Hunt and Handoo, 2009). The species that is most often associated with damage in bell pepper is the Southern Root-Knot Nematode, *M. incognita* (Di Vito et al., 1985; Di Vito et al., 1992; Thies et al., 2008). In California, RKN in bell pepper have traditionally been controlled through a pre-plant application of fumigant nematicides. Prior to the phase-out in 2015 because of its negative impact on the ozone layer (Fuller et al., 2008), the broad-spectrum fumigant methyl bromide was used in California bell pepper production to control weeds, soil-borne fungi, and nematodes. In 1995, 220,000 kg methyl bromide was used on 1,012 ha, by 2005 this had decreased to 18,700 kg on 104 acres, and in 2010 only 76 kg were applied on 0.4 ha (CDPR, 2021). Currently, 1,3-dichloropropene (55,950 kg on 450 ha), metam-sodium (304,555 kg on 1331 ha), metam-potassium (140,921 kg on 593 ha), and chloropicrin (16,784 kg on 147 ha) are the fumigants that are used in California bell peppers (CDPR, 2021). However, because of the negative effect of these fumigants on the environment there is increasing pressure to limit the use of these compounds. In a recent report by the California Sustainable Pest Management Workgroup (CDPR, 2023), the goal is to eliminate the use of priority pesticides by 2050. Although priority pesticides are not (yet) specifically identified in the report, it is likely that soil fumigants will fall in this category.

With the phase-out of methyl bromide and continuing pressure to limit the use of other fumigant nematicides, there has been renewed interest in the development of more sustainable and environmentally more benign non-fumigant nematicides. Fluensulfone (tradename Nimitz 480 EC; a.i. 48%, ADAMA Agricultural Solutions Ltd., Raleigh, NC) belongs to the fluoroalkenyl thioether group (Kearn et al., 2014) and was registered as a nematicide in California for use on fruiting vegetables in 2015, and for use on root and tuber vegetables in 2020. Fluensulfone has a “caution” label and a 12-hour re-entry interval. Consequently, there are no handling restrictions and less strict personal protective equipment requirements than for fumigant nematicides. Furthermore, unlike with fumigant applications, a fumigant management plan and non-treated buffer zones are not needed with a fluensulfone application (Castillo et al., 2018). Fluensulfone is applied as an incorporated drench or through chemigation at least seven days prior to seeding or transplanting at rates of 1.96 to 3.93 kg a.i. per ha. In field trials on a variety of vegetable crops including carrot (*Daucus carota*), cucumber (*Cucumis sativus*), eggplant (*Solanum melongena*), squash (*Cucurbita sp*.), potato (*Solanum tuberosum*), sweet potato (*Ipomoea batatas*), lettuce (*Lactuca sativa*), and tomato (*Solanum lycopersicum*) fluensulfone reduced root galling and/or soil population densities of RKN second stage juveniles (J2) compared with untreated control plants (Oka et al., 2009; Becker et al., 2013; Dickson and Mendes, 2013; Oka et al., 2013; Morris et al., 2016; Becker et al., 2019; Ploeg et al., 2019; Peiris et al., 2021). Although the exact mode of action of fluensulfone is unknown, it is a true nematicide that kills nematodes and its activity is irreversible (Oka and Saroya, 2019). Kearn et al. (2014) studied the effect of fluensulfone on *Caenorhabditis elegans in vitro* and concluded that it dramatically and negatively impacted embryotic development, egg-hatching, locomotion, and feeding, ultimately leading to mortality. Furthermore, they demonstrated that the mode of action was different from other non-fumigant nematicides such as aldicarb and ivermectin (Kearn et al., 2014). Fluensulfone seems particularly effective against RKN, while having only very minor effects on non-target organisms such as honeybees or earthworms (Oka and Saroya, 2019). With RKN, it affected host finding, nematode locomotion and mobility, and egg survival and hatching (Oka et al., 2009; Oka et al., 2013; Oka and Saroya, 2019; Oka, 2022).

Several studies on the systemic activity of nematicides, mostly focusing on oxamyl, were done in the 1970s (Rich and Bird, 1973; Taylor and Alphey, 1973; Gowen, 1977; Santo and Bolander, 1979), but this approach has not been adopted on a large scale under field conditions. The first report on the systemic activity of fluensulfone on RKN was by Oka et al. (2011). They reported that a single foliar spray of peppers with a fluensulfone solution at 3g/liter, 2 to 7 days prior to inoculation with *M. incognita* J2 significantly reduced the galling index and the number of nematode eggs compared with a water-only spray (Oka et al., 2011). They concluded that the systemic activity of fluensulfone was an advantage over other nematicides, but they also mentioned that the adverse phytotoxic effects of a foliar spray with fluensulfone on crops, such as tomato, may limit its applicability (Oka et al., 2011). Systemic nematicidal activity of fluensulfone against *M. incognita* on tomato was also reported by Morris et al. (2012) (2016) after a foliar spray application of young transplants, but this was accompanied by phytotoxic effects. The treatment was phytotoxic to eggplant, but not to cucumber or squash. However, on these crops systemic nematicidal activity was not observed (Morris et al., 2012; Morris et al., 2016). They concluded that phytotoxicity and systemic activity of fluensulfone differs among different crops (Morris et al., 2012; Morris et al., 2016).

The objective of this study was to evaluate the systemic activity and phytotoxic effects of fluensulfone used as a foliar application on transplants of different pepper cultivars and other vegetables crops, to determine if this could be a viable treatment option to protect vegetable transplants from early RKN infection prior to planting them in nematode-infested soil.

## Materials and Methods

### Nematode inoculum

A *M. incognita* race 3 population, originally isolated from infested cotton in the San Joaquin Valley, CA, was multiplied on pot-grown tomato plants in a greenhouse. Eggs were extracted from heavily infested tomato cv. Red Cherry roots by shaking them for 3 min in a 0.5% NaOCl solution, washing them over one 60 μm pore-size sieve, and then collected on two stacked 25 μm pore-size sieves (Hussey and Barker, 1973). The resulting egg suspension was carefully poured over a filter paper over a dish with tap water and placed at 27ºC. Hatched second-stage juveniles (J2) were collected daily from the third day onwards and kept at room temperature with a slow air flow bubbling through the J2 suspension. After 7 days, the number of J2 in the suspension was counted at 40x magnification under a dissecting microscope and the concentration was estimated.

### Crop cultivars tested

Tomato cv. Daniela (Osborne Quality Seeds, Mt. Vernon, WA), eggplant cv. Black Beauty (Park Seed, Greenwood, SC), cantaloupe (*Cucumis melo*) cv. Durango (Seminis, Oxnard, CA), bell pepper cv. Baron (Seminis, Oxnard, CA), chili pepper cv. Thai Dragon (Reimer Seeds, Saint Leonard, MD), and bell pepper cv. Sweet Mini Pepper (Sunworld, Bakersfield, CA) were used in one or more of the trials. All crops were seeded in seedling trays with potting mix (Sunshine Mix 5, Sungro, Vancouver, Canada) in a greenhouse at 27 ± 4 °C under natural light conditions and used when plants had 3 to 4 true leaves.

### Systemic activity and phytotoxicity

In a first set of experiments, seedlings of all crop cultivars at the 3 to 4 true leaf stage were removed from the seedling trays, the root systems gently washed with tap water to remove the potting mix, transplanted into 1 liter plastic pots filled with steam-sterilized sand (93% sand, 4% silt, 3% clay, pH 7.1) and then placed on a greenhouse bench for a week. All pots were then removed from the greenhouse into the shade, avoiding direct sunlight on the leaves. Plants were subjected to one of four treatments: 1) foliar spray with tap water (=untreated control), 2) foliar spray with 3 g/liter fluensulfone, 3) foliar spray with 6 g/liter fluensulfone, 4) foliar spray with 12 g/liter fluensulfone, and 5) a soil drench treatment. The amount of fluensulfone added to each pot for the soil drench treatment was based on a pot surface of 78.5 cm2 and the application rate of 2.8 kg fluensulfone per ha. To each pot, 100 ml of a 0.022 g/liter fluensulfone solution was added, which was sufficient to completely saturate the soil in the pot.

Before spraying, the soil of each pot was covered around the plant stem with a layer of plastic and three layers of paper to prevent contact between fluensulfone and the soil surface. For spraying, a pressurized hand-held sprayer was used delivering a fine mist, and spraying was stopped when the leaf surfaces were saturated with liquid. Each treatment had five replicates. Plants were allowed to dry for 10 minutes, paper layers and plastic sheet were removed, and pots were placed in a random order on a greenhouse bench not allowing the plants to touch. Two days after the treatments, all pots were inoculated with 2,000 *M. incognita* J2 by adding 1 ml of a suspension containing 500 J2/ml to each of four shallow holes around the base of each plant. Pots were watered though an automated drip system, and each pot received 5 g of Osmocote 17-6-10 controlled release fertilizer (Scotts-Sierra Horticultural Products Co, Marysville, OH) 3 days after nematode inoculation. Plants were visually examined for phytotoxic effects of the treatments 2, 7, and 14 days after applying the treatments. Phytotoxic effects were indexed on a 0–5 scale according to 0 = no symptoms, 1 = <20% of leaf surface with damage only visible on youngest leaves, 2 = 20–40% of leaf surface with damage and minor necrosis on the older leaves, 3 = 41–60% of leaf surface with damage and moderate necrosis on the older leaves, 4 = 61–80% of leaf surface with damage and major necrosis on the older leaves, and 5 = 100% of leaf surface with damage (dead plant).

Six weeks after nematode inoculation, all plants were carefully removed from the pots, and the root systems were gently washed free of soil with tap water. The plants were cut at the soil level, and the fresh and dry shoot weights of each plant were determined. The root systems were weighed and visually examined for the presence and severity of galling and indexed on a scale from 0 to 10, representing the presence of root galling on 0 to 100% of the root system. RKN J2 were extracted from each root system in a misting chamber for 5 days (Niblack and Hussey, 1985) and counted at 40x magnification under a dissecting microscope. The number of J2 per gram root and the reproduction factor (Rf) were calculated (where Rf = Pf/Pi, with Pi = the initial inoculum level (J2) and Pf = the final recovery of J2) (Thies, 2011). The experiment was done three times.

### Nematicidal activity in the rhizosphere

To test if a foliar application of fluensulfone had a nematicidal effect in the root rhizosphere, tomato cv. Daniela and pepper cv. Baron seedlings at the 3 to 4 true leaf stage were removed from the seedling trays, and the root systems were gently washed with tap water to remove the potting mix. Plants were placed flat on a table with the leave surface facing upwards and the roots were covered with two layers of filter paper and a sheet of plastic, leaving only the leaves exposed. There were 4 foliar spray treatments, applied as before: a spray with tap water (untreated control, and a spray with fluensulfone at 3, 6, or 12 g/liter. The leaves were allowed to air-dry for 10 min, an absorbent filter paper was then placed on the leaves for another minute, and plants were then transferred to 100 ml plastic vials containing 50 ml of water. Vials were wrapped with aluminum foil and plants were supported with an aluminum foil lid wrapped around the stem. The vials were placed in a random order on a table in a greenhouse, and two days later 1 ml of a suspension containing 500 *M. incognita* J2 was added to each vial. Five days later, the plants were removed from the vials and the number of dead and live J2 were counted in three 3 ml samples from each vial. J2 that were straight, not moving after prodding with a fine needle and with a disintegrated body content were considered dead. The average of the ratio dead/live J2 from the three subsamples was used for statistical analysis. Each plant-treatment combination had five replicates, and the experiment was done three times. The average greenhouse air temperature during the experiments was 23.3 (range 21.0–26.6), 27.8 (range 23.2–35.8), and 25.9ºC (range 23.8–38.9) in experiments one, two, and three, respectively.

### Timing of systemic movement of fluensulfone

To determine the time between a foliar spray with fluensulfone and the appearance of nematicidal activity in the rhizosphere, two repeated experiments were done. Tomato cv. Daniela and bell pepper cv. Baron were sprayed with fluensulfone (6 g/liter) as described above. A water-only spray served as a control. Immediately after transferring the plants to the vials, 1 ml of a suspension containing 500 *M. incognita* J2 was added to each vial. The plants were removed from the vials 2, 12, 24, 72, and 120 hr after the treatment and the nematodes in the vials were collected, examined, and J2 mortality in the suspensions was evaluated as described above. Each plant-treatment combination had five replicates, and the experiment was done three times.

### Statistical analysis

Effects of repeated experiments (1, 2, or 3), treatment, and the interaction between experiment and treatment on fresh and dry shoot weights and on nematode root infestation levels were analyzed using ANOVA procedures. Treatment means were separated using an LSD-test. For nematode infestation levels, data were transformed by x^1^ = log10 (x + 1) before analysis. For analysis of the ratio dead/live J2, data were transformed by arcsin (√x) before analysis. Non-transformed data are shown. Normality of fresh and dry shoot weight data and of transformed nematode infestation level data was examined using the Shapiro–Wilk test and through examination of Q-Q plots. Effects of the treatments on the categorical variables: the degree of phytotoxicity (scale 0–5) and the degree of root-galling (scale 0–10), were analyzed using the non-parametric Kruskal–Wallis test. The R software package was used for statistical analysis (R Core Team, 2022).

## Results

### Plant growth and phytotoxicity

Analysis of variance showed that with almost all crops tested, there was a significant interactive experiment × treatment effect on the fresh and dry shoot weights. Therefore, results were analyzed separately for each experiment. Shapiro–Wilk tests and examination of Q-Q plots showed that fresh and dry shoot weights for individual experiments and crops were normally distributed. For each of the crops the treatment effects on fresh and dry shoot weights were very similar, and therefore, only effects on dry shoot weights are shown.

Tomato fresh and dry shoot weights were affected by the treatments in each experiment. In two of three experiments, plants from the untreated control and the drench treatment resulted in higher fresh and dry shoot weights than plants sprayed with 6 or 12 g/liter fluensulfone. Averaged over the three experiments, fresh and dry shoot weights were significantly reduced by each of the spray treatments (Table 1). Fresh shoot weights were reduced by 24%, 45%, and 70%, and dry shoot weights were reduced by 38%, 63%, and 85% in the 3, 6, and 12 g/liter fluensulfone sprays, respectively.

**Table 1:** Average (n=5) dry shoot weight (± SE) of three vegetable crops treated with a fluensulfone spray at three rates (3, 6, 12 g/liter) or applied as a drench. Plants were evaluated 6 weeks after treatments in three replicated experiments.

**Crop**	**Treatment**	**Exp 1**	**Exp 2**	**Exp 3**	**Average**
Tomato cv. Daniela	UTC	74.1±6.0 a^1^	58.5±10.1 a	24.1±0.9 a	52.2±6.7 a
	3 g/liter	55.8±5.5 ab	19.7±4.1 b	19.5±1.6 b	32.5±5.1 b
	6 g/liter	35.4±8.5 c	6.2±1.7 b	14.4±1.7 c	19.5±4.3 c
	12 g/liter	14.2±5.6 d	3.7±0.2 b	4.3±1.6 d	8.1±2.3 d
	Drench	50.8±5.8 bc	64.2±10.5 a	24.9±0.2 a	46.6±5.7 a
*P*-value		<0.001	<0.001	<0.001	<0.00 1

Eggplant cv. Black Beauty	UTC	31.6±4.9 a	38.8±7.8 a	17.5±1.3 a	29.3±3.7 a
	3 g/L	3.7±2.0 c	-^2^	9.6±0.7 b	6.7±1.1 b
	6 g/L	13.1±4.5 bc	-	3.0±0.8 c	8.0±2.2 b
	12 g/L	-	-	-	-
	Drench	22.8±2.7 ab	37.1±7.8 a	13.7±1.9 a	24.6±3.7 a
*P*-value		<0.001	0.88	<0.001	<0.001

Melon cv. Durango	UTC	22.2±4.9 a	24.4±0.6 a	15.8±1.7 a	20.5±1.9 a
	3 g/L	-	-	-	-
	6 g/L	-	-	-	-
	12 g/L	-	-	-	-
	Drench	18.4±3.6 a	32.1±4.0 a	18.8±0.7 a	22.5±2.3 a
*P*-value		0.55	0.14	0.15	0.47

1 Different letters within columns within the same crop cultivar indicate significant differences at the 95% confidence level (LSD- means separation).

2 -dead plant.

**Figure 1:**
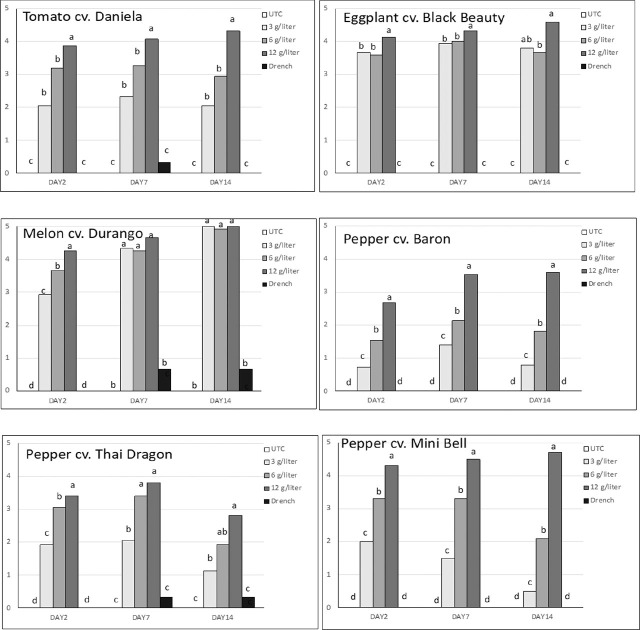
Average (n=15) phytotoxicity symptom ratings (scale 0-5: 0 = no symptoms, 5 = plant dead) of plants treated with fluensulfone as foliar spray at 3, 6, or 12 g/liter or applied as a soil drench at 5 pints/acre (0.022 g/liter) 2, 7, and 14 days after application. Water-sprayed plants served as untreated controls (UTC). Different letters above bars indicate significant differences at *P* ≤ 0.05, Kruskal–Wallis test.

The phytotoxicity ratings were significantly affected by the treatments in a similar way in the three replicated experiments. Strong phytotoxic effects were observed in the highest spray rate, intermediate effects in the 3 and 6 g/liter spray treatments, and very minor effects after the drench application (Fig. 1; Supplement Figure 1A).

A spray with the high rate (12 g/liter) of fluensulfone was highly phytotoxic to eggplant as none of the plants survived this treatment. In experiment two, a spray with the low or intermediate rate also resulted in plant death, but in experiment one and three, plants from these treatments survived. Dry shoot weights of the surviving eggplants were significantly lower than the control after being sprayed with 3 or 6 g/liter fluensulfone. The drench application did not significantly reduce the dry shoot weight (Table 1). On average, the fresh weights were reduced by 71% and 67%, and the dry shoot weights by 77% and 73% after the 3 and 6 g/liter spray treatments. Phytotoxicity ratings reflected the effects on shoot weights. Very strong phytotoxic effects were observed already two days after spraying plants with fluensulfone at all three rates (Fig. 1; Supplement Figure 1B).

**Figure 2:**
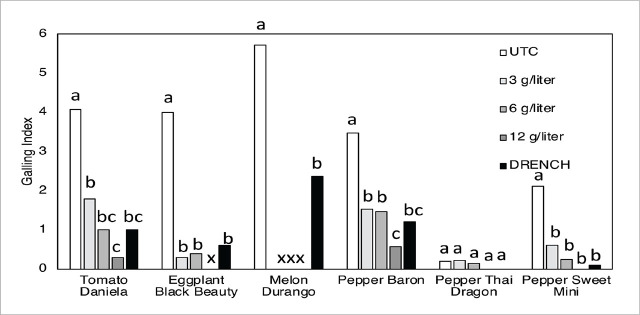
Average (n=15) root galling index (scale 0–10: 0 = no galling, 10 = 100% of roots with galls) of plants treated with fluensulfone as foliar spray at 3, 6, 12 g/liter, or applied as a 100 ml soil drench at 0.022 g/liter. Plants were inoculated with 2,000 *Meloidogyne incognita* J2 per 1 liter pot 2 days after treatments and evaluated 6 weeks after treatments. Water-sprayed plants served as untreated controls (UTC). Different letters above bars within the same plant cultivar indicate significant differences at *P* ≤ 0.05, Kruskal–Wallis test. x = missing (dead) plants.

Melon plants also reacted very strongly to fluensulfone foliar sprays. None of the melon plants that were sprayed with fluensulfone survived 6 weeks after nematode inoculation. However, pots with melon that were drenched with fluensulfone all survived, and the dry shoot weights of these plants were not affected by the drench treatments (Table 1). Phytotoxic symptoms on melon after fluensulfone sprays were already apparent two days after the treatments, and one week after these treatments most plants were dead or close to dying (Fig. 1; Supplement Figure 1C).

Pepper plants were slightly more tolerant to fluensulfone sprays than the other crops. Pepper Baron fresh or dry shoot weights were generally not significantly affected by a fluensulfone spray at the lowest rate of 3 g/liter. Effects of fluensulfone sprays on pepper Baron dry shoot weights were slightly different between the three replicated experiments. On average however, a significant reduction in shoot weights occurred after spraying the plants with fluensulfone at rates of 6 or 12 g/liter. The drench application did not affect Baron shoot weights (Table 2). Phytotoxicity symptoms increased with increasing fluensulfone rates of the spray application. The lowest rate resulted only in minor symptoms that did not progress over time, whereas the highest rate caused obvious phytotoxic effects. The drench application did not result in any phytotoxic effects (Fig. 1; Supplement Figure 1D).

**Table 2:** Average (n=5) dry shoot weight (± SE) of three pepper cultivars treated with a fluensulfone spray at three rates (3, 6, 12 g/liter) or applied as a drench. Plants were evaluated 6 weeks after treatments in three replicated experiments.

**Cultivar**	**Treatment**	**Exp 1**	**Exp 2**	**Exp 3**	**Average**
Baron	UTC	10.6±2.2 a^1^	7.8±2.8 ab	7.4±0.7 ab	8.6±1.2 a
	3 g/liter	8.4±3.4 a	2.3±0.8 c	8.0±1.3 ab	6.3±1.4 ab
	6 g/liter	5.1±1.5 ab	3.8±1.4 bc	6.5±0.8 b	5.1±0.7 bc
	12 g/liter	1.0±0.3 b	1.2±0.2 c	4.8±1.2 b	2.4±0.6 c
	Drench	5.9±1.5 ab	10.1±2.0 a	10.2±1.5 a	8.7±1.0 a
*P*-value		0.035	0.011	0.039	<0.001

Thai Dragon	UTC	8.3±1.4 a	16.0±1.6 a	4.1±0.7 a	9.5±1.5 a
	3 g/liter	4.8±1.2 a	4.3±0.7 c	9.7±1.7 a	6.4±1.0 bc
	6 g/liter	3.3±1.8 a	8.2±2.3 bc	3.7±1.3 a	4.9±1.1 c
	12 g/liter	3.1±1.3 a	7.4±1.8 bc	6.1±1.6 a	5.3±0.9 c
	Drench	7.9±2.4 a	10.1±1.0 b	8.2±2.1 a	8.7±1.1 ab
*P*-value		0.27	<0.001	0.08	0.02

Sweet Mini	UTC	3.5±0.9 a	-^2^	4.0±0.9 a	3.8±0.6 a
	3 g/liter	1.1±0.3 b	-	4.4±1.1 a	2.7±0.8 a
	6 g/liter	0.4±0.1 b	-	8.1±0.5 a	4.2±1.3 a
	12 g/liter	-	-	3.7±1.5 a	3.7±1.1 a
	Drench	2.2±0.9 ab	-	5.6±1.8 a	3.9±1.1 a
*P*-value		0.047		0.23	0.88

1 Different letters within columns within the same crop cultivar indicate significant differences at the 95% confidence level (LSD-means separation).

2 -dead plant.

Thai Dragon pepper shoot weights were only affected by the treatments in one of the three replicated experiments (experiment 2). Averaged over the three experiments; however, shoot weights were significantly reduced by the fluensulfone spray applications (Table 2). Phytotoxic effects occurred with all three spray applications, with the stronger effects after the high-rate fluensulfone sprays. Plants did, however, somewhat recover over time, as the average phytotoxicity rating of each spray application at Day 14 was lower than at Day 2 or Day 7 (Fig. 1; Supplement Figure 1E).

In the second replicate experiment with Sweet Mini peppers, all plants were lost due to an accidental herbicide spray. Therefore, data are from two replicated experiments only. Effects of the treatments on the shoot weights were different between the two experiments but averaged over the two experiments the treatments did not significantly affect the shoot weights (Table 2). There was a clear correlation between the rate of the fluensulfone spray and the severity of phytotoxic effects. The high rate resulted in severe symptoms, whereas the lower rate only caused minor symptoms which also decreased over time (Fig. 1; Supplement Figure 1F).

### Systemic activity

Because the treatments affected plant growth, the effects of the treatments on *M. incognita* infestation levels were corrected for plant root weight (number of J2/g root). Treatment effects on root galling (scale 0–10) were very similar between the three replicated experiments and are shown as averages of the three experiments (Fig. 2).

On tomato, all fluensulfone treatments significantly reduced the root nematode infestation levels compared with the untreated control. Overall, the strongest reductions resulted from the 12 g/liter foliar spray (99% reduction) and the drench application (97%) (Table 3). Results on root galling were similar, with significant reductions resulting from each of the fluensulfone treatments (Fig. 2).

**Table 3:** Average (n=5) *Meloidogyne incognita* root infestation (J2/g root ± SE) of three vegetable crops treated with a fluensulfone spray at three rates (3, 6, 12 g/liter) or applied as a drench. Plants were evaluated 6 weeks after treatments in three replicated experiments.

**Crop**	**Treatment**	**Exp 1**	**Exp 2**	**Exp 3**	**Average**
Tomato cv. Daniela	UTC	153±33 a^1^	25±4 a	81±16 a	86±18 a
	3 g/liter	38±18 b	2±1 b	11±1 b	18±7 b
	6 g/liter	3±2 c	1±1 b	9±3 b	5±1 b
	12 g/liter	1±0 c	2±1 b	0±0 d	1±0 d
	Drench	4±1 c	0±0 b	3±1 c	2±1 c
*P*-value		<0.001	<0.001	<0.001	<0.001

Eggplant cv. Black Beauty	UTC	650±87 a	24±5 a	249±85 a	308±79 a
	3 g/L	8±4 b	-^2^	0±0 b	4±2 b
	6 g/L	5±3 bc	-	0±0 b	2±1 b
	12 g/L	-^2^	-	-	-
	Drench	0±0 c	2±1 b	1±0 b	1±0 b
*P*-value		<0.001	<0.001	<0.001	<0.001

Melon cv. Durango	UTC	196±84 a	61±9 a	795±127 a	371±99 a
	3 g/L	-	-	-	-
	6 g/L	-	-	-	-
	12 g/L	-	-	-	-
	Drench	37±12 b	13±2 b	313±37 b	129±39 b
*P*-value		0.014	0.0014	0.0025	0.0163

1 Different letters within columns within the same crop cultivar indicate significant differences at the 95% confidence level (LSD- means separation). Data were transformed by x^1^ = log10 (x + 1) before analysis, non-transformed data shown.

2 -dead plant.

On eggplant, all fluensulfone treatments strongly reduced root nematode infestation levels, although some of the foliar spray treatments also resulted in plant death. On average, foliar sprays at 3 and 6 g/liter and the drench treatment all reduced root infestation levels by ≥98% compared with the untreated control (Table 3). These treatments were similarly effective in reducing root galling on eggplant (Fig. 2).

Only untreated and drench-treated melon plants survived. On average, the drench treatment significantly reduced root infestation by 65% and the average root galling index from 5.7 in the untreated control to 2.4 in the drench treatment (Table 3 and Fig. 2).

There were some differences between the three replicated experiments regarding the efficacy of the fluensulfone treatments on reducing nematode infestation in Baron peppers but on average the 12 g/liter foliar spray and the drench treatment were most effective reducing infestation levels by 99% and 98% compared to the untreated control (Table 4). These two treatments were also most effective in reducing Baron root galling (Fig. 2).

**Table 4:** Average (n=5) *Meloidogyne incognita* root infestation (J2/g root ± SE) of three pepper cultivars treated with a fluensulfone spray at three rates (3, 6, 12 g/liter) or applied as a drench. Plants were evaluated 6 weeks after treatments in three replicated experiments.

**Cultivar**	**Treatment**	**Exp 1**	**Exp 2**	**Exp 3**	**Average**
Baron	UTC	1,119±125 a^1^	160±39 a	404±52 a	561±117 a
	3 g/liter	128±45 b	45±19 bc	31±14 b	68±19 b
	6 g/liter	149±52 b	11±7 cd	25±5 b	62±23 b
	12 g/liter	6±3 c	4±2 d	11±4 c	7±2 c
	Drench	0±0 d	35±6 ab	3±1 c	12±5 c
*P*-value		0.035	0.014	0.039	<0.001

Thai Dragon	UTC	0±0 a	0±0	28±17 a	10±6 a
	3 g/liter	44±44 a	0±0	2±2 a	16±15 a
	6 g/liter	12±12 a	0±0	0±0 a	4±4 a
	12 g/liter	0±0 a	0±0	0±0 a	0±0 a
	Drench	0±0 a	0±0	0±0 a	0±0 a
*P*-value		0.63	NA	0.33	0.36

Sweet Mini	UTC	80±23 a	-^2^	881±285 a	525±196 a
	3 g/liter	8±3 b	-	80±34 ab	44±29 b
	6 g/liter	5±4 bc	-	5±4 b	5±4 bc
	12 g/liter	-^2^	-	0±0 b	0±0 c
	Drench	0±0 c	-	2±2 b	1±1 c
*P*-value		0.047	NA	0.23	<0.001

1 Different letters within columns within the same crop cultivar indicate significant differences at the 95% confidence level (LSD- means separation). Data were transformed by x^1^ = log10 (x + 1) before analysis, non-transformed data shown.

2 -dead plant.

Nematode infestation levels in Thai Dragon peppers were low and variable even in the untreated control plants (Table 4), indicating that this cultivar is a poor host for *M. incognita*. Consequently, there were no significant differences among the treatments. Correspondingly, only very minimal galling occurred on Thai Dragon pepper roots, and there were no significant treatment effects (Fig. 2).

Nematode infestation levels in Sweet Mini Pepper roots were also variable between experiment one and three, with on average 80 and 881 J2 per g root in the untreated control plants, respectively. Averaged over the two experiments, however, all fluensulfone treatments significantly reduced J2 root infestation levels by more than 90% (Table 4). Galling on Sweet Mini Pepper roots was minor even in the untreated control plants, but on average all fluensulfone treatments were equally effective in reducing root galling (Fig. 2).

### Nematicidal effect of fluensulfone in the rhizosphere

**Figure 3:**
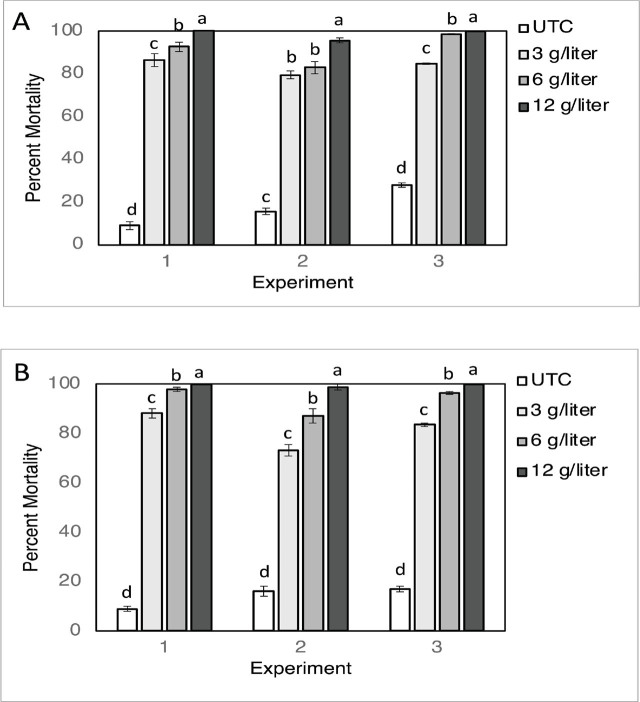
Average (n=5) percent mortality, 5 days after treatments, of *Meloidogyne incognita* J2 in a water suspension surrounding the roots of tomato (A) or pepper (B) plants treated with a foliar spray application of fluensulfone (3, 6 and 12 g/liter). UTC = untreated control. Three replicated experiments were done. Error bars are ±SE. Different letters above bars indicate significant differences within the same experiment at *P* ≤ 0.05, LSD-test. Percentage mortality data were transformed by arcsin (√x) before statistical analysis. Non-transformed data are shown.

Foliar applications with fluensulfone had strong nematicidal effects in the water surrounding the roots 5 days after the applications were made. The arcsin (√x) transformed data (ratio dead/live J2) satisfied normal distribution requirements (Shapiro–Wilk test: P > 0.05). Although ANOVA analysis indicated a significant interaction between the treatments and the replicated experiments, the results were very similar between the three replicated experiments for both the tomato cv. Daniela and pepper cv. Baron test plants. With tomato, the *M. incognita* J2 mortality levels in the untreated control plants ranged between 9% and 28%, whereas the mortality in the water surrounding the roots of the fluensulfone-treated plants was significantly higher, ranging between 79% and 100%. Results with pepper were very similar, with a significantly higher J2 mortality in the water surrounding the roots of the fluensulfone-treated plants compared with the untreated plants. Except for tomato in experiment 2, each increase in the fluensulfone rate resulted in a significant increase in J2 mortality (Fig. 3).

**Figure 4:**
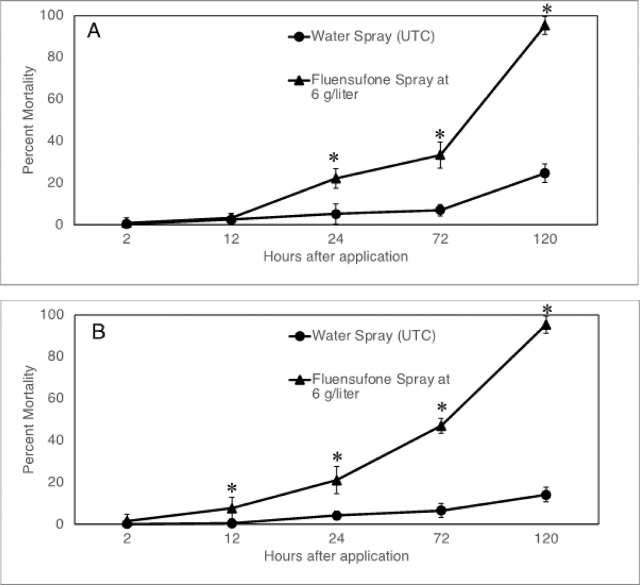
Average (n=15) percent mortality of *M. incognita* J2 in a water suspension surrounding the roots of tomato (A) or pepper (B) plants treated with a foliar spray application of 6 g/liter fluensulfone at different time intervals after application. UTC = untreated control. Error bars are ±SE, * indicate significant differences within the same time interval at *P* ≤ 0.05, LSD-test. Percentage mortality data were transformed by arcsin (√x) before statistical analysis. Non-transformed data are shown.

Statistical analysis of the results of the subsequent time-series experiments with tomato and pepper showed that the factor experiment (1, 2, 3) or the “experiment x treatment” interaction was not significant (P>0.05). Therefore, results are presented as averages of the three repeated experiments (n=15). Results showed that treatment effects on J2 mortality became apparent as soon as 12 hr (pepper plants) and 24 hr (tomato plants) after the applications were made. At these times, the mortality of *M. incognita* J2 in the root solution of fluensulfone-treated plants was significantly greater than in the water-sprayed control plants. Differences between these two treatments increased over time, and at 120 hr after application, the average J2 mortality in fluensulfone-treated tomato or pepper plants was 95%, whereas mortality in untreated tomato or pepper plants was 25% and 14%, respectively (Fig. 4).

## Discussion

Oka et al. (2012) reported the possible systemic nematicidal activity of fluensulfone in pepper. In this study, the range of crops was expanded to examine if fluensulfone may also be used when applied as a foliar spray in other important vegetable crops. This study provides some very useful information on the potential use of fluensulfone as a foliar spray in crops susceptible to RKN. The first set of experiments showed that all crop cultivars suffered phytotoxic damage after a foliar spray with fluensulfone. Melon Durango was particularly sensitive, as none of the plants survived a fluensulfone spray at any of the three rates and damage was already severe one week after treatment. Eggplant was also sensitive as the high rate always, and the lower rates sometimes resulted in plant death, and the damage was severe as soon as two days after treatment. Tomato was moderately sensitive as the fresh and dry shoot weights significantly decreased with increasing fluensulfone rates. Pepper, particularly cv. Baron was somewhat tolerant, as the lower rates generally did not affect shoot weights and resulted only in mild or moderate symptoms. Results with pepper cv. Thai Dragon and cv. Sweet Mini were variable, but these two cultivars somewhat recovered from the lower and medium rate applications. Thus, the sensitivity of crops towards foliar sprays with fluensulfone differed among the different crops, which agrees with earlier studies (Oka et al., 2011; Morris et al., 2012; Morris et al., 2016). However, our results are different from those by Morris et al. (2016). They evaluated effects of foliar sprays with fluensulfone at the same rates as in our trials (3, 6, and 12 g/liter) and did not observe major effects on tomato vigor or dry shoot weights but did observe some phytotoxicity on eggplant (Morris et al., 2016). Surprisingly, as in our experiments melon cv. Durango was the most sensitive crop cultivar to the phytotoxic effects of a fluensulfone spray, Morris et al. (2016) reported no effects of similar treatments on the vigor or dry shoot weight of the cucurbits cucumber or squash.

All fluensulfone spray treatments significantly reduced the nematode root infestation in tomato, eggplant, and pepper cv. Baron. In melon, none of the sprayed plants survived, in pepper cv. Thai Dragon infestation levels were low even in the untreated controls, and pepper cv. Sweet Mini spray treatments also almost always reduced nematode infestation. Significant reductions in nematode infestation of roots after foliar spray treatments with fluensulfone were also observed by others, even when fluensulfone was compared to foliar applications of oxamyl and fenamiphos (Oka et al., 2011; Oka et al., 2012; Morris et al., 2016). Morris et al. (2016) obtained significant reductions in root nematode infestation and root galling only in one (tomato) of four crops tested and only at the 6 and 12 g/liter rates and the 12 g/liter rate, respectively. Furthermore, the levels of reductions were much less than in our trials, as they only achieved significant reductions in nematode infestation levels in tomato roots of approximately 33% and 45% after the 6 g and 12 g/liter foliar spray, respectively. In our trials these levels for tomato were 95% and 99%, respectively. Oka et al. (2011, 2012) evaluated the effects of foliar spray applications on pepper, and their results show highly significant reductions in root galling and nematode infestation levels after fluensulfone sprays at 3 or 6 g/liter that agree with our results on pepper cv. Baron.

Because phytotoxic effects on tomato Daniela and pepper Baron after fluensulfone foliar sprays were relatively moderate, and the spray treatments in both these crop cultivars significantly and consistently reduced nematode infestation, these two crop cultivars were selected for subsequent experiments on systemic root-exudation. The nematicidal activity of fluensulfone applied as a foliar spray at rates of 3, 6, or 12 g/liter on tomato cv. Daniela or pepper cv. Baron plants, was translocated into the water surrounding the roots of these plants, as evidenced by the high mortality of the RKN J2 in the water surrounding the roots. Furthermore, this effect was significant as soon as 24 hr after the treatment and increased as time progressed. This is the first report that demonstrates that the systemic activity of fluensulfone applied as a foliar spray results at least in part from the active ingredient being exuded by the roots into the surrounding environment. Oka et al. (2012) pointed out a lack of evidence showing that root exudates of fluensulfone-treated plants were nematicidal, thereby inhibiting nematode invasion of the roots. The results from this study provide evidence that this is true and that such root exudates are likely to have a nematicidal effect on the nematodes in the soil rhizosphere. However, whether this effect is similar in a water suspension as in this study, compared to a field situation where nematodes move through a water film surrounding soil particles, remains to be determined. Similar observations with the systemic nematicide oxamyl were made by Taylor and Alphey (1973), Potter and Marks (1976), and Wright et al. (1980) who concluded that a compound with nematicidal activity was exuded into the soil surrounding roots, or root-rhizosphere soil of cucumber plants treated with a foliar spray of oxamyl. The time of translocation of the nematicidal effect into the roots of 24 hr as observed in our trials, was similar to that obtained by Rich and Bird (1973) who concluded that oxamyl activity was also translocated into the roots of tomato and cotton in 24 to 48 hr.

We conclude that a foliar spray of transplants of melon Durango or eggplant Black Beauty with fluensulfone is not recommended as a RKN management strategy as it results in severe damage to the transplants. A foliar spray of tomato Daniela at a rate of 6 g/liter resulted in significant reductions in nematode infestation with only moderate phytotoxic effects. Pepper Baron and Sweet Mini plants were more tolerant towards foliar fluensulfone sprays, and a spray at 3 g/liter resulted in only mild phytotoxic effects, while still resulting in (mostly) significant reductions in nematode infestations. Pepper Thai Dragon was a poor host for the RKN used (*M. incognita* race 3) and also moderately sensitive to a foliar fluensulfone spray at 3 g/L. The effectiveness of a foliar spray of transplants with fluensulfone as a nematode management strategy under field conditions remains to be evaluated.

A soil drench of the pots in which transplants were planted, was mostly very effective in reducing nematode root infestation, while not resulting in any significant phytotoxic effects. Drenching seedling trays with transplants was not included as a treatment in this study but may offer another option for the early protection of transplants from nematode infection once planted in the field. Nematode control by a foliar spray with a systemic nematicide has several advantages over a “regular” soil application, such as a reduction in the amount of product needed, ease of application, protection of the entire root system, and a lower risk of soil and/or groundwater pollution. The latter may become increasingly important, as fluensulfone has been listed among pesticides with active ingredients that can be considered as PFAS (per- and poly-fluoroalkyl substances) (Anonymous, 2025). These substances have been receiving a lot of attention recently as compounds that are very persistent and mobile in the environment and potentially toxic to humans (US EPA, 2019).

In this study, a soil drench of fluensulfone proved to be very effective in controlling *M. incognita*. Others obtained similar results in field trials with carrots, cucumber, cantaloupe (Westerdahl et al., 2014), and tomato (Westerdahl et al., 2015).

Some questions by Oka et al. (2012) still remain unanswered. A concern is the toxicity of fluensulfone applied to the plant foliage to humans, because the fate of the compound on the plant is not known. Also, nothing is known yet about the mode of fluensulfone translocation from foliage to roots, such as its translocatable form in the plant, the concentration of nematicidal molecules in root cells and root exudates and the effect on nematode behavior in the rhizosphere.
